# The role of Clinical Officers in the Kenyan health system: a question of perspective

**DOI:** 10.1186/1478-4491-11-32

**Published:** 2013-07-17

**Authors:** Patrick Mbindyo, Duane Blaauw, Mike English

**Affiliations:** 1 Kenya Medical Research Institute Centre for Geographic Medical Research Coast-Wellcome Trust Collaborative Programme, P. O. Box 43640–00100 GPO, Nairobi, Kenya; 2 Centre for Health Policy (CHP), School of Public Health, Faculty of Health Sciences, University of the Witwatersrand, P.O. Box 1038, Johannesburg 2000, South Africa; 3 Department of Paediatrics, John Radcliffe Hospital, University of Oxford, Oxford, UK; 4 Nuffield Department of Medicine, John Radcliffe Hospital, University of Oxford, Oxford, UK

## Abstract

**Background:**

Despite the increasing interest in using non-physician clinicians in many low-income countries, little is known about the roles they play in typical health system settings. Prior research has concentrated on evaluating their technical competencies compared to those of doctors. This work explored perceptions of the roles of Kenyan non-physician clinicians (Clinical Officers (COs).

**Methods:**

Qualitative methods including in-depth interviews (with COs, nurses, doctors, hospital management, and policymakers, among others), participant observation and document analysis were used. A nomothetic-idiographic framework was used to examine tensions between institutions and individuals within them. A comparative approach was used to examine institutional versus individual notions of CO roles, how these roles play out in government and faith-based hospital (FBH) settings as well as differences arising from three specific work settings for COs within hospitals.

**Results:**

The main finding was the discrepancy between policy documents that outline a broad role for COs that covers both technical and managerial roles, while respondents articulated a narrow technical role that focused on patient care and management. Respondents described a variety of images of COs, ranging from ‘filter’ to ‘primary healthcare physician’, when asked about CO roles. COs argued for a defined role associated with primary healthcare, feeling constrained by their technical role. FBH settings were found to additionally clarify CO roles when compared with public hospitals. Tensions between formal prescriptions of CO roles and actual practice were reported and coalesced around lack of recognition over COs work, role conflict among specialist COs, and role ambiguity.

**Conclusions:**

Even though COs are important service providers their role is not clearly understood, which has resulted in role conflict. It is suggested that their role be redefined, moving from that of ‘substitute clinician’ to professional ‘primary care clinician’, with this being supported by the health system.

## Introduction

Recent research has shown that non-physician clinicians (NPCs), a form of mid-level worker, may be a viable solution to bringing physician-type services closer to people that need them while long-term solutions to recruiting and retaining qualified health professionals especially in rural areas are sought [1-6]. Mid-level workers (MLWs) are healthcare providers who have received less training, have a more restricted scope of practice than professionals, and are accredited by their countries’ licensing bodies [1]. In Kenya, NPCs are currently known as Clinical Officers (COs), of whom more exist (COs number 1,353 compared with medical officers and specialists that number 491) at district level [7]. The Kenyan CO cadre has two subgroups; general COs (RCOs) and specialist COs (SCOs; these are COs who have undertaken further specialist training in a medical discipline). COs are regulated by the Clinical Officers Council, an institution mandated under the Clinical Officers Act (CAP 260) to oversee their training, registration and licensing in Kenya. This body provides guidance on illness to be handled by COs, requirements for continuous professional development for continued registration, and issues licenses for private practice. However, the Council is not seen to have much influence over day-to-day CO roles, which is left to the hospitals where they work.

Available literature on COs suggests that they play distinct and important roles in the day-to-day delivery of health services [8,9]. However, what their roles are and how these are perceived by COs and others is rarely described. Understanding CO roles is important as the literature supports a link between an individual’s role in an organization and their attitudes towards work that, if negative, can result in dysfunctional behaviours [10]. To understand the role issues facing COs, we draw on work describing professional tensions between doctors and nurses. Tensions between nurses and physicians arise because of overlapping roles, nurses desire for collegiality, and changing role relationships as nurses achieve increased levels of education [11]. In addition, research also shows that nurse practitioners can do some of what doctors do, usually to the greater satisfaction of patients [12]. Nurses experiencing role conflict (described as inconsistent job obligations) and role ambiguity were reported to be less satisfied with their jobs which in turn negatively influenced their job performance and also lowered their organizational commitment [13].

Much of the research undertaken on non-physician clinicians, however, focuses on whether they are able to perform technical tasks (Caesarean section, provision of antiretroviral therapy (ART) and so on) previously the preserve of physicians [14-16]. The positive results from this work have supported the growing interest in such cadres as a solution to physician shortages in many sub-Saharan countries. However, this focus on technical aspects of the role ignores the non-technical but equally important aspects of work [1,2,17]. As this cadre likely plays specific roles in the system there is need to understand this cadre specifically. The one previous study that did examine non-technical aspects of the work of clinical officers reported that COs experience significantly greater levels of dissatisfaction with their jobs and with their profession compared to other cadres [6]. Yet in these studies, there is little description of the roles played by COs that might give insight into what, in their routine practice, results in these feelings of dissatisfaction.

This report seeks to fill this gap by describing the roles played by COs in Kenya, a country with decades of experience using COs within the health system. Here, roles refer to the actions and activities assigned to, required of or expected of a person or a cadre in a substantive organizational position [18,19]. This is performed by examining the formal prescriptions of CO roles and goes further to explore perceptions of the role holders (COs) as well as their colleagues and supervisors in the frontline. A comparison of views from government and faith-based hospital respondents is employed to achieve a broader understanding of CO roles.

## Methods

**Figure 1:**
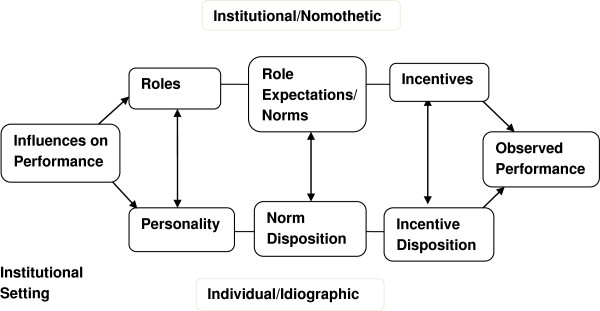
The modified nomothetic-idiographic model.

The conceptual framework used to explore CO roles was the Getzels and Guba model [20] which conceptually delineates the interaction between institutions and individuals. Although there are many other possible models [21,22] this was chosen for its applicability to the work proposed. While mainly applied in education to understand issues such as role conflict among teachers [23], teacher burnout [24], roles of special administrators[25], the Getzels – Guba model has also been applied to other areas such as evaluation of educational personnel [26], and safety leadership in university laboratories [27], among others (Figure 1).

The model proposes that any social system (a health system, a hospital, a hospital department, and so on) has two interdependent but interacting dimensions, the nomothetic (or normative) and the idiographic (or personal) [20,28]. The institution (nomothetic dimension) consists of formally espoused roles, role expectations/norms and incentives (and sanctions) [28]. Within the institution are individuals (idiographic dimension) with certain personalities, need dispositions and motivational dispositions [20]. By occupying roles (job positions) in the institution, they help it to achieve its goals. The literature suggests that performance is improved if employees’ goals are well aligned with those of the organization [29,30]. This paper therefore explores a nomothetic prescription of CO roles that helps define how COs are expected to act to meet health system goals and contrasts this with respondents perceptions of their roles. Perceived roles are however one expression of idiographic and institutional influences. Tools for the study were developed following a nomothetic-idiographic approach as well as the literature review, and were finalized after being piloted in two non-study hospitals.

Qualitative methods were used that included document analysis, individual in-depth interviews, and observation in areas where COs work at the study sites. Document analysis began the process of acquiring data on COs based on first, a review of literature on COs, then a review of national and hospital level that described aspects of CO functioning in the health system or hospital. Thus, documents such as Government policy documents on COs (CO Act, CO schemes of service, performance appraisal form, norms and standards for service delivery, and so on) and hospital documents (routine hospital data on numbers of staff and clients, performance appraisal forms, CO job descriptions, and so on) were analyzed for information on the nomothetic aspects of CO work [31]. A semi-structured interview guide was used and included questions such as: ‘What do you regard as being the functions of COs in the health system?’ and, ‘Are COs prepared to carry out their roles?’, and so on. The interview guide was developed based on issues emerging from document analysis and literature review, and was finalized based on findingsfrom a pilot study in two hospitals that were excluded afterwards. Participant observation was conducted at the same time with interviews and used to examine the daily experiences and practices of COs in their work context in order to understand the factors that influence their performance [32]. This included observing health worker behaviours and practices such as time to report to work and, time spent in queues for example waiting in line in the outpatients department (OPD) to see a clinician. We also participated in informal conversations or meetings with the understanding that what was observed was data. There was no instance of refusal to observe. Observations were recorded immediately after and reviewed at the end of each day. Observations were also informed by issues arising from interviews.

Study sites were six hospitals located in three Kenyan provinces purposefully selected to represent the diversity of hospital settings in which Kenyan COs work. Three were public hospitals (H2, H3, H5) and three were faith-based (H1, H4, H6) and are characterized in Additional file 1: Table S1. Public hospitals were purposefully selected based on anecdotal evidence showing variation in ‘performance’ and the fact that they were based in large rural towns. Faith based hospitals that provided a comparative aspect to the study were selected on the basis of being part of Christian Health Association of Kenya (CHAK), which has been requesting the government to pay for the deployment of COs to work in these facilities as they serve a significant proportion of Kenya’s rural population. While non-Christian hospitals such as those run by the Muslim faith could have been chosen, they were deemed less suitable because they are few in number and rarely employ COs. Within hospitals, we explored three settings: OPDs, vertically supported clinics (VCs, for example HIV clinics) and specialist clinics run by COs (SCOs,for example ophthalmology clinics). Few COs work in inpatient areas in district hospitals in Kenya. The focus on district hospitals was driven by the fact that they host over 50% of government employed COs [33]. Within the 6 hospitals, a total of 68 interviews were conducted. Respondents included COs (both general and specialists; =40), MOs and pharmacists (4), hospital management (comprising of hospital CEOs, medical officers in charge, hospital matrons, hospital administrators, human resources officers, CO supervisors; 9) and nurses (11) in hospitals who were available in the period spent in each hospital. Further key-informant interviews were conducted with policymakers (senior Ministry of Health Officials whose tasks involve them with CO issues) and the Secretary General of the Kenya Association of Clinical Officers. Policymakers were interviewed in the early stages of the study to inform the issues to be explored and after undertaking the pilot study. This dataset that also included data from document reviews and notes from participant observation was explored using three different perspectives to produce three complementary reports, the first focusing on roles of COs, the second examining their norms of performance, and a third exploring notions around incentives for their performance.

All interviews were transcribed into Microsoft Word 2007 (Microsoft; Redmond WA, USA) and then imported into NVIVO 8 software (QSR International, Brisbane, Australia) categorized by source (hospital) and type of interview (key informant, in-depth or data from participant observations). Coding into themes was performed iteratively using the directed content analysis approach that starts with theory or relevant research findings to help derive the initial codes [34]. Initially, coding was performed separately by hospital to enable a description of the specific setting and to enable hospital level differences to be explored. Where appropriate all codes from the six hospitals were combined into one. Following from the coding, three sets of analyses were performed to interrogate the data and inform its reporting. First, data was explored whether there was coherence between the institutional and individual levels in understanding of roles. Second, data were also used to illuminate and characterize the roles COs play in day-to-day work. Third, data were interrogated to explore whether there were differences in the roles performed by COs that could be attributed to features of the clinical settings where COs work or the hospitals (government or faith-based).

Ethical approval was sought and received from the National Ethics Review Committee housed in the Kenya Medical Research Institute and the University of Witwatersrand’s Committee for Research on Human Subjects.

## Results

### Nomothetic perspectives on CO roles

Unlike many other countries, in Kenya COs are subject to professional and regulatory oversight outlined by the Clinical Officers Act of 1989 [35]. However, although this act refers largely to duties of the clinical officer cadre, the current foundational basis for the roles that all COs carry out in the Kenyan health system is derived from COs schemes of service (SOS) of which two exist. The first was developed in 1994 [36] and a more recent one published in 2009 [37]. This information was supplemented with data drawn from other government and hospital policy documents. The duties and responsibilities that are expected to be carried out by COs in the revised CO scheme of service published in 2009 are outlined in Table 1. This articulates the type and complexity of services to be undertaken by both COs and SCOs and the key result areas and performance expectations for each activity assigned to COs.

**Table 1:** Duties, key result areas and performance standards for junior Clinical Officers (COs)

**Duties**	**Key Result Areas**	**Standards of performance**
Patient care and management	A. Attending and treating patient ailments in an Outpatient/Inpatient department in a hospital, health center or dispensary.	A) Documentation of history taking, physical examination, investigation and diagnosis of patients ailments and management
	B. Counsel clients on treatment and compliance to treatment	B) Clarity of investigation form(s) and correct interpretation of results
		C) Clear documentation of prescriptions and follow-up of clients
		D) Record all cases seen daily
Planning and conducting community health care activities	A. Identify community health needs	A) Documentation of community health needs
	B. Plan and conduct community health activities	B) Documentation of interventions undertaken to address community health needs
	C. Develop reports for community health activities	
	D. Establish community networks through community healthcare workers and community own resource persons (CORPs) (for COII and above)	
Training, counseling and guiding clinical students attached to the hospital/health center	A. Identify training needs of students and staff	A) Documentation of training plan for students and staff in the facility
	B. Develop and conduct trainings and counseling for students in the facility	B) Report of counseling and training program for students and staff in the facility
		C) Orientation of students on clinical practice/areas and maintenance of their records
		
Supervising and counseling a small number of staff engaged in routine patient care and giving support and health education to patients	A. Develop support supervisory plan	A) Documentation of support supervision and on the job training of staff
	B. Identify training and counseling needs of staff	B) Provide on the job training and counseling of staff

Tables 1 and 2 outline the duties, key result areas and performance standards for work performed by COs mainly in the public sector. It should be noted that though the schemes of service detail all issues related to CO work, they do have an almost exclusive focus on task related issues especially related to patient care and management. An overwhelming focus on task issues, as shown in Tables 1 and 2, could result in neglect of non-task issues such as collegiality, resulting in poor work relationships.

**Table 2:** Summarized duties of mid and senior level clinical officers (COs)

**Mid-level COs**	**Senior level COs**
**Duties outlined in Table **1** plus:**	Management of Clinical Services involving:
A. Training of community health workers	A. Formulation of clinical services policies
B. Secretary to Health Committees	B. Maintenance of clinical standards and ethics
C. Management of clinical services in a Provincial/ District hospital or health center	C. Deployment of clinical officers in the Ministry
D. Curriculum development, its implementation and evaluation	D. Training and development of clinical officers
E. Partnership for development that involves liaising with division heads on health services.	E. Staff performance appraisal
	F. Planning, implementation and Supervision of curriculum development;
	G. Evaluation of training program
	H. Research

As COs increase in seniority, they take on additional duties as shown in Table 2. Seniority here is taken to mean COs who have worked in hospitals for more than 5 years and/or have specialized in an area of medicine to become specialist COs.

Reinforcing the CO schemes of service are other policy documents such as the Norms and Standards for Health Service Delivery [38]. The Norms and Standards document concentrates on three general areas of service provision thought to be key [38]:

– history taking, examining, diagnosing, treating and follow-up of patients and clients in medical health institutions and the community; (2)

– offering specialized services for COs with higher diplomas such as ear, nose and throat (ENT), ophthalmology/cataract surgery, pediatrics and child health, anesthesia, orthopedics, epidemiology, lung/skin, reproductive health^b^ , dermatology and venereology at all levels of health delivery and program

– providing community health services including health education and promotion, disease control, prevention and management; follow-up, data collection, disease surveillance, monitoring and evaluation, standards and quality assurance, home based care and research.

(NB Reproductive health is a new area of specialization among COs. It is still facing resistance from MOs specialized in obstetrics and gynecology.)

In addition to performing mainly clinical duties as shown above, COs also work as heads of health centres or dispensaries, as district COs, or as coordinators of special clinical services /program at provincial level, for example tuberculosis (TB)or child health, which means that they are expected to perform managerial tasks in addition to clinical duties. The scheme of service also includes more policy related responsibilities for the most senior COs such as management of in the administration of the national department/division responsible for the cadre including; formulation of clinical services policies; and maintenance of clinical standards and ethics. COs mainly work at health centers through to provincial hospitals in the system though there are some who work in national referral hospitals [39]. A considerable number of COs work at the district level and in particular at the more than 200 district hospitals [33]. At this level, both Government and faith-based hospital (FBH) sites proscribed very similar duties for COs mainly related to patient care.

### Idiographic perceptions on CO roles

The following section examines respondent opinions on the roles of COs in the sites visited. In exploring issues facing COs, many stated that they were familiar with the 1994 scheme of service. On probing further, few COs reported actually having seen this and some reports indicated that COs were actually referring to the legislative document (CO Act) as the basis of their work. In fact, much of the information on their roles was acquired from their peers, their supervisors, or hospital management. Almost all respondents were unaware of the updated 2009 scheme of service.

Consistent with the COs scheme of service, there was a general consensus among respondents that the CO role involved the provision of physician type health services to walk in patients in the Kenyan health system, as shown by the quotes below.

‘We see all patients unless we have difficult cases which we refer to other hospitals’.

Hospital CO in charge, H3

‘Clinical officers play a very important role in the provision of … services at even level four and level five *(district and provincial hospitals)* because they run the casualties, they run the outpatients and then they run specialized clinics’.

Policymaker 2

In dispensary or health center facilities that provide primary care and outpatient services, a CO is often the overall manager, running the entire facility. Managing lower level facilities however appears to be a challenging position as the CO, often alone as a clinician, has to take responsibility for decisions for which they are perhaps not legally supported. A district CO (DCO) in H2 supported this saying that ‘…The problem is that the CO might know the procedure, but is not allowed by law to do it’. Despite this challenge, some respondents saw it as an invaluable learning opportunity especially for younger COs who did not have much experience working in hospitals.

At district and provincial hospitals respectively, CO roles were often expanded as COs with appropriate specialist training worked as SCOs in their area of qualification. SCOs generally were then able to restrict their scope of practice to their area of specialization. They had greater autonomy that included being the ‘lead’ clinician when treating patients referred to them and also performed minor surgical procedures. As described in the quote below, these officers routinely work in specialist clinics (ENT, ophthalmology, and so on) or in chest and lung clinics that offer treatment of tuberculosis supported by the National HIV/AIDS control programme (NASCOP). However, any CO (general or specialist) who has undergone training to offer ART could work in clinics providing HIV/AIDS treatment (that is comprehensive care clinics). In addition, SCOs also mentored students and interns attached to the hospital while those specialized in chest and lung diseases were required to supervise lower level facilities that offered TB services. The following two quotes from one respondent highlight these issues.

‘You have eye problems? Go to the eye clinical officer. You have an orthopedic problem? Yes. Go to the orthopedic clinical officer. I mean, really, when you look at that, we are not saying that the general clinical officer…yes, he has a lot to do, he’ll treat common ailments, but he’ll give the specialist clinical officer, his work!’

Policymaker1

‘And they are very important! In a country like Kenya where you have very few doctor-specialists, they are very important because they are the same guys who actually play a big role in these specialized areas’.

Policymaker1

Senior level COs such as the CO in charge of all COs in a hospital also performed functions such as workforce management including organizing shifts, rotation through departments, appraising performance, organizing continuous medical education sessions and others.

‘I manage COs in the hospital, prepare the duty roster, assign working areas, schedule leave and outreaches, and handle outbreaks’.

Deputy CO in charge, H3

Functions that COs rarely performed include carrying out outreach services for example through screening patients in the community to identify those who need interventions or providing health education. These issues appear to be neglected perhaps due to high patient loads, staff shortages, financial constraints or misperception of their roles, an issue described below.

#### Respondent representations of CO roles

While the predominant representation of a CO in literature is that of a ‘physician substitute’ suggesting a temporary fixture, actual interviews with study respondents revealed five common understandings of COs that are presented as ‘images’ of COs. The existence of these images contrasts with the idea ‘substitution’, suggests permanence within the health system and a tension between formal (policy or managers) and individuals notions of who a CO is.

#### Filter

‘Every patient who comes to the hospital must pass through the hands of a clinical officer in the outpatient department after which they are discharged home, directed to special clinic, referred to the medical officer’s office, or admitted to the wards’.

CO FGD, H2

‘We deal with the patients that they refer to us…they are a sort of filter’.

OPD MO, H2

‘Clinical officers will provide the first referral level for outpatients, managing the clients as referred by the nurses. This will largely be at the outpatients’.

MOH (2006:10): Norms and Standards for Service Delivery [38]

Viewing COs as a ‘filter’, an idea also described in formal prescriptions of COs [38], was the predominant image reported by respondents. The ‘filter’ image interprets COs role to be the gate keepers of the health system suggesting a narrow, mechanistic understanding of their place in the health system. The norm across all sites was that the CO acts like a patient ‘sieve’: any patient visiting the hospital would first be seen by a CO then if necessary, be referred to a doctor, admitted to the wards or be referred for specialist treatment as shown by the quotes above.

#### Backbone of the physician health service

‘As you are aware, the clinical officers in Kenya form the backbone of health services particularly in the rural areas’.

SCO Ophthalmology, H2

‘There is a big number of patients who will not be treated if clinical services were to be offered only by doctors. In delivering services to those people, I think the clinical officer has been vital in the healthcare system to reach those people’.

Consultant, H1

The image of the ‘backbone’ was also commonly reported by all categories of health workers and came up when respondents were probed about the value of CO’s services to the country. This was reinforced by recognition that they provided physician type services in rural areas where there were few doctors and where they work for longer periods in a single area.

#### Face of the hospital

Study respondents felt that hospital users translated what COs did and how well they performed their work to be what the hospital stands for and what is to be expected therein. As such, there is pressure for COs (and other cadres) to ‘conform’ to the expectations that the hospital management has of them, an issue given impetus by the implementation of a civil service performance improvement initiative. These issues are highlighted in the quotes below.

‘The OPD is the image of the hospital. Whatever service is given reflects on the hospital. The first clinician to see patients is the CO. So, if our service is good, it determines how well patients are treated. If poor, the hospital gets the blame’.

CO in charge, H5

‘COs are the first-line medics of the nation. They are more at risk of getting infectious diseases than other cadres, yet they are the least considered’.

Policymaker 3

For the CO however, being the face of the hospital has its risks. As first line medics of the nation, COs feel that they were at a higher risk of getting infections than other cadres. They also felt that they manage high numbers of patients without being given risk allowances (available to other cadres) that reflect the risky nature of their work.

#### Sandwich

‘They assume they are a sandwich between doctors and nurses.’

Medical Superintendent, H2

‘They feel pressured from every side’.

SCO Pediatrics, H4

The ‘sandwich’ image acts as a counterpoint to the other three images described previously and was mainly reported by hospital management and COs. This notion highlights pressures arising from inter-cadre relationships that could be better in some sites. The notion of being ‘sandwiched’ refers to COs feeling that they were positioned between doctors who have hierarchical authority and nurses who have numerical authority. The notion here is that COs can be done away with despite the preceding ideas that suggest their importance. Additionally, While there is pressure for COs to accomplish the tasks that are allocated to them, many CO felt that their job attracted much pressure that was not understood by other cadres and managers which had negative implications for their performance.

#### Primary healthcare clinician

‘The clinical officer is one of the key service providers in this country especially at the primary healthcare level’.

Policymaker 2

‘I might say that from the experience I have had with the clinical officers that I have met, that they are relatively well trained in dispensing healthcare at the primary level and I think they are relatively well trained in recognizing limitations that there are’.

Consultant, H1

The image here amalgamates positive aspects of the previous images to present one of an individual who is trained to do specific tasks and is responsible for providing these at the primary level. It supports the notion that COs are well suited to providing preventive or primary healthcare services and if well utilized, might begin to reduce the number of patients seeking care from higher level hospitals (from district hospitals to national teaching and referral facilities). While this image fits well with the ailments that COs in private practice are allowed to treat (based on prescriptions in the CO Act), it is interesting that COs did not refer to themselves as specifically filling a primary care clinical role.

### CO roles in different work settings

This section examines the differences in CO roles that could be attributed to their places of work, that is faith based (FBH) or Government (GOK) hospital settings. The following table summarizes differences between three clinical work settings as reported by general and specialist COs. These issues could be seen to be motivating or de-motivating factors associated with those clinical settings (Table 3).

**Table 3:** Comparison of Clinical Officer (C)O work settings

**CO type**	**Outpatient department**	**Specialist clinics**	**Vertical clinic**
**General COs**	•Filtering patients see all patients seeking services in the hospital	•Seen to have easy work as clinicians see patients already diagnosed	•Focus either on tuberculosis (TB) or HIV/AIDS
	•Refer difficult cases to specialist clinics or to senior clinicians	•Seen to have a lighter workload, thus clinicians working there seen not to be working hard	• Thus, work follows pre-defined job procedures and guidance on performing these is available
	•Have little or no autonomy about their work		•Resources always available as externally funded
	•Work is done in shifts (morning afternoon or night), with night duties being disliked		•Have motivating working environments as most sites were recently constructed
	•Outpatients always has a heavy workload		
	•Is described to have poor working relationships with other cadres but not in all sites		
	•Shortages of supplies to do work reported in some sites		
**Specialist COs**	•Resistance to working here resulting in friction with hospital management due to shortage of COs in some sites for outpatients	•See referred and walk-in patients and perform minor and sometimes major procedures;	
	•Seen to be a place of escape from the heavy workloads and night duties associated with outpatients	•Refer difficult cases to senior clinicians	
		•Can admit and follow-up patients in inpatient wards	
		• Have more autonomy to determine work procedures	
		• Lighter workload compared with outpatient department	
		•Good working relationships with colleagues	

Interview data from COs and others suggests that both FBH and GOK facilities accorded COs similar general roles which coalesced around patient care and management for those working in the outpatient department. However, COs from FBH settings reported greater management efforts at role clarification (through access to Consultants and regular meetings with management) as compared to their GOK counterparts who relied on experiential learning from college training or peers. Of interest, very few CO specialists were found working in FBH facilities as these institutions generally preferred a doctor-managed system. However, the increasing high cost of recruiting and retaining MOs had made them begin to recruit COs to run their outpatient departments (with support from MOs) while MOs focussed their effort on covering the FBH’s inpatient departments and surgical theatres. Only CO Anesthetists worked in FBH facilities due to the shortage and high cost of maintaining MO Anesthetists.

Variation in the roles played by COs in work settings within hospitals, that is the OPD, specialist CO clinics and vertically supported clinics (VCs, providing HIV/AIDS care) were also seen. CO roles in GOK hospital OPDs generally focused on patient care and management and tended to function as a stand-alone cadre. FBH OPDs generally supported their COs by either providing an MO or Consultant in the OPD to provide backup to COs. In difficult cases, GOK COs admitted the patient for further care or referred them to a SCO or consultant. SCOs generally focussed on taking care of patients within their area of specialization. This fact and often physically distinct clinical rooms in OPDs accorded them some autonomy. In VCs that provided care for patients with TB and/or HIV/AIDS, the roles were very clear as the program provided set rules and guidelines for COs working in such environments.

### Limited CO career mobility

There was considerable congruence between formal prescriptions of COs roles within hospital settings. However there were also differences in how CO job descriptions differed with those perceived or enacted by COs suggesting a tension between institutional expectations and those of the individuals within them. These tensions are seen as forms of conflict between the nomothetic and idiographic dimensions. While the new CO scheme of service provides for a relatively broad role, their role in practice was largely limited to first-line patient care and management as captured by most of the images referred to earlier. This setting of limits can restrict professional growth and thus recognition and many COs felt that their contribution to health service delivery was not acknowledged. The lack of recognition was seen in the paradox between being called ‘the back bone of the physician service’ or ‘face of the hospital’, yet experiencing constraints such as poor promotion or poor career progression prospects exist that have resulted in a perception of the existence of a ‘glass ceiling’ in their careers, as shown by the following two quotes by the same respondent.

‘There appears to be a ‘glass ceiling’…upward movement is very poor, unless one moves out of CO medicine into other areas’.

Policymaker 3

‘For those who go for the additional training to specialize, the qualification does not result in an increase of their salary. They come back and work at the same job group as they left. This is quite de-motivating’.

Policymaker 3

The lack of upward progress was also reported by a policymaker who felt that the present forms of advancement moved COs away from clinical medicine to other areas, an issue that was not desirable. Being accorded the appropriate recognition and appreciation would also perhaps address COs perception that doctors are often given credit for work that COs do.

The role conflict seen in the study (defined as the situation where a person must adhere at the same time to two or more conflicting or contradictory sets of expectations [40] is related to SCOs who generally saw their specialist skills as making them different from general COs. In their opinion, specialist skills gave them the ability to work with consultants (medical officers with postgraduate training) or independently where no consultants were available. Thus, efforts by hospital managers to get SCOs to work in OPDs due to the shortage of RCOs were resisted with some respondents feeling that their elevated status was not being recognized.

‘When you go for specialized training you are supposed to practice within your area of expertise although you are not supposed to forget general medicine’.

SCO ENT, H5

‘The care given is more specific, the workload is less, but there is friction between us and the administration because they want us to work in the OPD. But who will see our patients? They won’t help when we have an overload’.

SCO Pediatrics, H3

## Discussion

Study respondents generally saw the work of a CO as important in providing physician type services in district hospitals and rural areas in Kenya. Despite the presence of a CO scheme of service that describes CO roles, no respondents in the rural hospitals reported having not seen it. Thus, CO roles were reported on the basis of those required of them by their supervisors. This perhaps explains the variability in images of COs seen in respondent descriptions of CO roles that ranged from ‘face of the hospital’, ‘sandwich’, to ‘filter’ and so on, that generally coalesced around patient care and management. Other CO functions as outlined in the scheme of service were not implemented*.* Three tensions arising from differences in perceptions of CO roles were reported and are discussed below.

The first tension arises from whether there is agreement between institutional prescriptions of COs with those of the COs themselves and others. The study found that CO roles from a nomothetic (institutional) perspective were derived mainly from the CO Act [35] and subsequently amplified in their schemes of service and several other policy documents [37,38] . These define the CO role as a permanent one that basically provided primary healthcare physician services ranging from patient management and care to training and mentorship and management oriented services for health committees at their places of work. The cadre is seen to provide important clinical services in rural areas where few physicians work. For example, the National Human Resources for Health Strategy (2009 to 2012) planned to increase the number of doctors by 893 over a 4 year period while that of COs was much higher at 2,185 over the same period [41]. However, their role as enacted was largely limited to diagnosing and treating walk-in patients (functioning in a ‘filter’ role) which was also reiterated in formal policy document [39]. For example, while health education and promotion activities are important in reducing incidence of preventable diseases that account for a large percentage of the cases handled by COs, these are rarely carried out in the community by public hospitals. People visiting public hospitals especially in the mornings get to hear health education and promotion lecture on an issue of interest. However, these campaigns are undertaken if there is an outbreak of an epidemic. The main divergence from such roles was seen in the work performed by specialist COs who occupied specific niches related to their areas of specialization, niches which provided greater autonomy and offered them the chance of isolating themselves from roles generally associated with the CO cadre.

Second was the evidence of role conflict arising from what institutions (hospitals) want and what COs, especially specialist COs, believe they can or ought to do. In some hospitals, specialist COs were being asked to work in the outpatient department, something they rejected and felt was undermining their special professional status. The predominant focus on task aspects of patient care (further described in another report) and management in a role as a ‘filter’ also reinforces the idea of COs as ‘physician substitutes’ [1] or even subordinates. This role was not necessarily liked by COs who argued that they were a separate cadre and were able to undertake a variety of professional roles if given the opportunity. To achieve this health systems will need to go beyond their current focus on CO technical roles to develop these other aspects that literature suggests will impact on their performance [10].

The third tension relates to role ambiguity, referring to tensions arising from limited knowledge about CO qualifications and understanding of their prerogatives and scope of practice by those who manage or work with COs [42]. This can be seen from different images that describe different ways in which COs are perceived which in turn can result in inconsistent role demands on COs. The result, in addition to the creation of uncertainty among COs of what they should do, is that there is poor recognition and appreciation of COs, an issue also reported in Tanzania [1] and Malawi [3]. This is despite the fact that they carried out work similar to that performed by doctors and by so doing, reduced the workload that potentially could accrue to doctors. Considering the influence of role ambiguity and role conflict on the work of COs, we posit the need for additional research on these issues. Other important issues reported by COs to constrain their role within the system include the limited career progression opportunities (the glass ceiling), findings also reported from Malawi [3].

An additional issue is the need to consider the effect of the internal conflict within health workers that arises from the tension between the need to be altruistic and at the same time self-interested so as to make a living from and create an identity for their profession. The effect of these issues is little described in literature perhaps because of a pervasive mechanistic view of COs as passive health workers who are not engaged with the complexity of negotiating who they are and what they do. Much of this relates to their low hierarchical position and the fact that senior positions in many ministries of health are held by doctors [5,9].

Our findings have some limitations. Many COs work in primary healthcare settings often as single clinicians together with nurses and in the absence of any physicians. Their responses may have been somewhat different. Even at the hospital level the study only visited six hospitals in Kenya, a relatively small sample of the more than 200 that now exist, although we did purposefully include 3 different provinces and both FBH and GOK hospitals. Further, the data reported here are essentially descriptive. We did not aim nor try to explore the historical or political reasons that have resulted in both the formally espoused roles of COs and how these are defined by the realities of practice within health systems. Rather we focused on attempting to characterize the end result of these processes that have shaped COs roles within Kenya over several decades. Interestingly, different cadre responses were consistent on the issues facing COs although it was not a formal intention to contrast them. Useful lessons for other countries implementing or considering deploying CO equivalents might be gained by further work in this area.

## Conclusions

While COs are acknowledged to provide an important curative services to walk in patients and basic surgical procedures in hospitals in rural Kenya. However, there is an overwhelming focus on their ability to perform technical tasks of patient care and management as outlined by their responses and emphasized by policymakers and hospital management. This is seen to have resulted in a neglect of other aspects of CO roles that include health education and promotion in the community, which perhaps would serve to reduce the number of patients visiting hospitals with preventable illnesses. This is despite the fact that their training and scope of service allows for much broader roles than are currently enacted. Further, respondents expressed varied perceptions of who they thought COs were suggesting that not much is known of them. This additionally serves to hinder their career opportunities with reports of poor recognition and appreciation accorded to their role. Issues arising from this include role conflict and role ambiguity for specialist COs and lack of recognition for what COs do. Thus, there is need to go beyond policy statements to promote and develop the roles that COs play in health systems that might promote job satisfaction among COs and motivate them to help deliver broader health system goals. This argues for an appreciation of COs that goes beyond the idea of ‘substitute physician’.

## Competing interests

The authors declare that they have no competing interests.

## Authors’ contributions

PM carried out the study under supervision of DB and ME. DB and ME participated in the data analysis. PM drafted the initial manuscript that was subsequently revised by DB and ME. All authors read and approved the final manuscript.

## Supplementary Material

Additional file 1: Table S1. Summary of hospital characteristics. Click here for file
