# Supplementary material for: The role of Clinical Officers in the Kenyan health system: a question of perspective
Source: Hum Resour Health. 2013 Jul 17;11:32. doi: 10.1186/1478-4491-11-32 (PMC3724708; doi:10.1186/1478-4491-11-32)
Supplement: Additional file 1: Table S1. — Summary of hospital characteristics. [file 1478-4491-11-32-S1.pdf]

Table S1. Summary of Hospital Characteristics

Hospital	No. of Beds	No. of Staff	No. of Theatres	Training Centre	Geographical Location
**H1** **(FBH)**	220 (+60 cots)	564; 35 MOs ; 2 Kenyan consultants; 25 foreign consultants	5; planned expansion to 9	Nursing; Internship centre for COs & MOs; train registrars	47kms from Nairobi. Rural. Agricultural area; highly influenced by Nairobi. Technical support from the African Inland Mission
**H6** **(FBH)**	280	8 consultants; 8 MOs; 7 COs.	3	Plans underway to begin nursing courses with Catholic University of East Africa	Located in Nairobi's Langata suburb, next to expansive Kibera slums. Affiliated to the Catholic Church- mainly supported by patients fees
**H4 [43]** **(FBH)**	275 (+16 cots)	3 resident MOs; 4 consultants; various local and international volunteers	1	Internship centre for COs; Accredited CPD centre (KMPDB); Training centre for student physicians via formal collaboration with	209 kms from Nairobi. Rural. Highly agricultural. Support from the United Methodist Church; infrastructure, personnel and equipment support though MOMS; various local and international

				KEMU;	NGOs
**H2**<br><br>**(GOK)**	306 (+17 cots)	Consultants – 11; MOs – 12; MO Interns – 7; RCOs – 21; CO Interns – 30;	2	Internship centre for COs & MOs; Meru MTC located next to hospital	174kms from Nairobi. Highly agricultural; location of national wildlife reserves. Urban centre.
**H5**<br><br>**(GOK)**	265 (+24 cots)	5 MOs, 12 MO Interns,10 Consultants,; 10 RCOs, 50 CO interns	3	Training centre for COs, Nurses, MOs, BSN,	39 kms from Nairobi, considered its satellite town; major highway connecting them; agriculture & industrial enterprises
**H3**<br><br>**(GOK)**	305 (+35 cots)		1		200kms from Nairobi. Semi-arid region. Rural. Location of wildlife conservancies & armed forces (including police) training bases. Livestock keeping, market farming

Source: http://www.ehealth.or.ke/facilities/facility.aspx?fas=13218
